# Optimizing Engagement with Digital Mental Health Resources Among Sexual and Gender Minority Users: Protocol for a Series of Microrandomized Trials

**DOI:** 10.2196/97126

**Published:** 2026-06-30

**Authors:** Meghan Romanelli, Theresa Nguyen, Kevin Rushton, John Marion

**Affiliations:** 1 School of Social Work, University of Washington, 4101 15th Ave NE, Seattle, WA, 98105, United States, 1 206-685-6948; 2 Mental Health America, Alexandria, VA, United States

**Keywords:** sexual and gender minority, digital mental health services, engagement, microrandomized trial, depression, clinical trial

## Abstract

**Background:**

Sexual and gender minority (SGM) populations experience significant mental health disparities, yet continue to face persistent barriers to care. Mental Health America (MHA) provides free, web-based screening and self-guided resources to millions of visitors, including tens of thousands of SGM visitors each year. Digital mental health (DMH) can facilitate access to mental health resources; however, engagement remains a central challenge that limits effectiveness. MHA faces similar engagement challenges, with preliminary analyses indicating that most visitors exit the website without accessing substantive mental health content pages. The QT-Digital Mental Health Engagement study uses an iterative approach to develop and test strategies for improving engagement with DMH resources among SGM users.

**Objective:**

This study provides the rationale and protocol for an iterative series of microrandomized trials testing theory-based engagement strategies embedded within the web infrastructure of a real-world DMH platform. The primary aim is to evaluate whether delivering engagement strategies increases SGM visitors’ naturalistic engagement with MHA resources.

**Methods:**

Eligible participants will be MHA visitors who complete the Patient Health Questionnaire-9 depression screening test in English and indicate on an optional postscreening survey that they live in the United States, are aged ≥14 years, and identify as LGBTQ+ (lesbian, gay, bisexual, transgender, queer, and other SGM populations). Participants will be randomized at 2 decision points embedded within the MHA Online Screening Program. At each decision point, participants will be randomized with equal probability (0.2) to receive no engagement strategy or an engagement strategy targeting 1 of 4 Health Action Process Approach behavioral determinants, including outcome expectancy, risk perception, self-efficacy, or SGM-specific barriers to engagement. The primary outcome will be proximal engagement with MHA content, operationalized at decision point 1 as click-through to a targeted Next Steps Content Page and at decision point 2 as click-through to any additional MHA content page. The primary analysis will estimate the marginal causal excursion effect of delivering any engagement strategy compared with the control.

**Results:**

This study received funding from the National Institute of Mental Health in June 2023 (K01MH131795). The protocol was approved by the single institutional review board at the University of Washington on December 3, 2025 (STUDY00017726). Data collection began on March 12, 2026, and is projected to end in July 2026. As of April 13, 2026, a total of 3878 participants have been enrolled, with 52% (n=2004) of participants identifying as SGM visitors aged 14 to 17 years.

**Conclusions:**

This protocol describes an iterative series of microrandomized trials designed to test theory-based engagement strategies within a real-world DMH platform where disengagement can occur within seconds. Results will identify which strategies show promise for improving naturalistic engagement among MHA’s SGM visitors and inform future refinement of engagement strategies and adaptive decision rules for optimizing engagement with DMH resources.

## Introduction

### Background

Sexual and gender minority (SGM) populations experience significant mental health disparities relative to their heterosexual and cisgender counterparts [1-5]. Depression, for example, is one of the most common, treatable mental health concerns [6], but SGM populations remain disproportionately impacted [1,7]. Research shows that the risk of depression among sexual minority populations ranges between 2 [8] and 3 [9] times that of heterosexual peers, with gender minorities reporting similar risks for clinical depression relative to cisgender counterparts [3,5]. Despite heightened need, SGM populations face persistent barriers to care, including those shared with the general population (eg, financial and geographic barriers) as well as unique to SGM care-seekers and shaped by minority stress processes (eg, discrimination and limited availability of affirmative providers) [10-15]. These barriers, especially those distinctive to the SGM care-seeking experience, can result in forgone care or difficulties with engagement in care that lead to significant unmet mental health needs [16,17].

Digital mental health (DMH) offers a promising solution to address barriers that restrict marginalized populations’ access to mental health care [18-20]. For SGM care-seekers, specifically, self-guided DMH may be an acceptable mode of service delivery with features that align with SGM care preferences (eg, privacy or anonymity, self-reliant and self-directed care, and remote access) [21-26]. However, engagement remains a central challenge that limits the effectiveness of DMH resources [27,28], including for SGM users [29]. In particular, self-guided DMH and DMH resources deployed in real-world settings are often used briefly or sporadically. Engagement patterns defined by low sustained use limit opportunities for behavior change and symptom improvement [30-32].

One strategy used to support engagement is the delivery of brief prompts or messages that direct users toward intervention content and encourage interaction at moments when engagement is most likely to decline [33-35]. However, most research on these messaging strategies has focused on push notifications delivered through mobile apps, often during structured trials. Less is known about whether similar approaches improve naturalistic engagement with real-world DMH platforms where disengagement is high and can occur rapidly. Furthermore, few studies have examined how analogous “pull” strategies embedded within website content may influence engagement as users navigate digital resources [36]. Emerging evidence from a large naturalistic trial on the Mental Health America (MHA) website suggested that tailoring messages and resources within a self-guided mental health platform can influence engagement behaviors, including among SGM users. However, effects varied across strategies, and some approaches introduced small engagement costs [37].

MHA is a national nonprofit organization that provides free, web-based mental health screening tools and self-guided resources designed to support individuals across the mental health continuum. The MHA website attracts approximately 7 million visitors annually and offers evidence-based screening instruments along with “Next Steps” resources, including psychoeducational articles, interactive self-help tools, peer support options, and treatment referrals. After completing a screening tool such as the Patient Health Questionnaire-9 (PHQ-9), visitors receive personalized results and are directed to recommended Next Steps resources. As these services are freely accessible and self-guided, MHA represents a high-fit platform for SGM care-seekers who often prefer private, self-directed help-seeking pathways.

The scale and naturalistic use of the MHA platform provide a unique opportunity to examine engagement with DMH resources under real-world conditions. Each day, hundreds of SGM visitors access MHA, representing a substantial proportion of the screening population. Despite the accessibility of MHA resources and strong reported intentions to seek information and self-help tools, engagement with self-guided DMH content remains brief for many visitors. This gap between stated intentions and observed engagement highlights an opportunity to test strategies designed to support visitors at key risk points of disengagement. Embedding an experimental design within this real-world setting allows for a systematic evaluation of which engagement strategies increase proximal engagement with DMH content, when they are most effective, and for whom. To address these questions, this study embeds a series of microrandomized trials (MRTs) within MHA’s Online Screening Program to experimentally test engagement strategies delivered at critical decision points and generate evidence needed to inform adaptive decision rules for optimizing engagement with self-guided DMH resources.

### Preliminary Work

As part of the overall grant funding, several phases of the QT-Digital Mental Health Engagement study were conducted prior to implementing the current MRT protocol. This work followed the discover, design, build, and test approach, a framework grounded in human-centered design [38]. This sequential research process aimed to (1) discover how SGM visitors engaged with DMH content on MHA’s website, (2) identify engagement barriers and treatment preferences to facilitate the design and build of tailored engagement strategies, and (3) provide an initial test of the strategies that target engagement.

This work used the Health Action Process Approach (HAPA) to frame our understanding of DMH engagement. HAPA is a behavior change framework that describes how individuals translate intentions into action. HAPA emphasizes immediate determinants of behavior that can be addressed in the moment, including outcome expectancies, perceived risk, self-efficacy, and barriers to and resources that support DMH engagement.

Preliminary analyses of screening and website metadata showed that approximately 63% of MHA visitors exited the site after viewing their screening results and did not visit any substantive content pages. Among those who engaged, time spent on content pages was typically brief. These patterns revealed distinct challenges with both initial and sustained engagement. To identify mechanisms underlying these engagement patterns, we linked behavioral metadata with a questionnaire assessing behavioral intentions and immediate determinants of behavior. Predictive models indicated that outcome expectancies, perceived risk, self-efficacy, and barriers such as feeling overwhelmed by action were among the strongest determinants of engagement. These findings suggested that engagement may be shaped by modifiable cognitive and motivational factors rather than by access alone.

To deepen this understanding, we conducted asynchronous online focus groups with SGM youth and adults to understand their experiences with DMH, including their journeys in accessing DMH resources, barriers to and facilitators of engagement, and tailoring and design preferences. Participants described a range of factors shaping DMH engagement that mapped onto HAPA behavioral determinants of engagement. While some concerns mirrored those of a general user population (eg, data privacy), others reflected experiences specific to SGM users. For example, they described fear of outing, exposure to stigmatizing content, and invalidation of identity. Participants described that they would be more likely to engage with DMH resources that signaled credibility with SGM communities, used nonjudgmental language, clearly communicated community involvement in creating or reviewing content, and incorporated recognizable visual cues, indicating that the content was safe and affirming for SGM users. These findings reinforced that engagement barriers may be amenable to design solutions that address HAPA behavioral determinants.

Guided by empirical findings and HAPA theory, we identified likely points of disengagement on the MHA website and developed brief engagement strategies designed to target behavioral determinants of engagement. Insights from focus groups informed the types of messages or visual cues most likely to influence SGM users’ outcome expectancies, perceived risks, self-efficacy, and perceived barriers to using DMH resources. Strategies were iteratively refined through both MHA and user feedback to enhance clarity, effectiveness, and feasibility within MHA’s existing web infrastructure.

### Microrandomized Trial

The MRT is an experimental design used to optimize interventions, including DMH. In a traditional randomized controlled trial, individuals are randomized once to an intervention or control condition. In contrast, an MRT repeatedly randomizes individuals to intervention components or no intervention at prespecified decision points (eg, points in time and places in the digital environment) where intervention components can be delivered to target a proximal outcome [39]. Therefore, MRTs effectively test both intervention timing and content. This repeated randomization also enables estimation of the causal effect of delivering a specific component on a proximal outcome measured shortly after each decision point and facilitates examination of whether that effect varies across time or participant characteristics.

In this study, we will embed an iterative series of MRTs within the MHA Online Screening Program to deliver engagement strategies as part of the standard website experience. In each MRT iteration, participants are randomized at 2 prespecified decision points where engagement strategies can be delivered within a single website session. These decision points represent empirically identified moments in the website flow where MHA visitors are at risk of disengagement. At each decision point, participants are randomized to either receive a HAPA-based engagement strategy or standard content. Although the number of decision points is constrained by the naturalistic website environment, repeated randomizations across these decision points allow estimation of the marginal causal excursion effects of the engagement strategies. This design makes it possible to determine whether and which engagement strategies are most effective at increasing naturalistic engagement in a real-world DMH setting. It also supports iterative refinement of strategy delivery (eg, which strategies are displayed, when they are delivered, and for whom they might be most effective) and the development of adaptive decision rules.

### Objective

The overall objective of this study is to test theory-based engagement strategies within the existing web infrastructure of a real-world DMH platform. Using a series of MRTs, engagement strategies designed to target HAPA’s immediate behavioral determinants will be delivered at 2 key decision points within the flow of MHA’s Online Screening Program to examine whether and which engagement strategies increase SGM visitors’ proximal engagement with MHA resources.

The primary aim is to evaluate whether delivering (vs not delivering) HAPA-based engagement strategies increases naturalistic engagement with MHA resources. Within this primary aim, we will also explore whether (1) the effectiveness of delivering (vs not delivering) engagement strategies differs across the 2 decision points and (2) the effects of delivering (vs not delivering) engagement strategies are moderated by time-invariant and time-varying factors.

The secondary aim is to examine whether engagement strategies targeting distinct HAPA behavioral determinants differ in their relative effectiveness in increasing naturalistic engagement with MHA resources. Within this secondary aim, we will also explore whether specific engagement strategy variations targeting the same HAPA behavioral determinant differentially impact proximal engagement.

## Methods

### Overview

This protocol was guided by the SPIRIT (Standard Protocol Items: Recommendations for Intervention Trials) guidelines (Checklist 1).

### Trial Setting

MHA’s Online Screening Program is a web-based program that provides access to free, confidential, and scientifically validated screening tools. Information gathered from the screening can help connect MHA visitors to resources, including articles about mental health and interactive self-help tools. This trial will enroll eligible visitors who access the PHQ-9 via MHA’s Online Screening Program during the trial period.

Engagement with MHA will be examined naturally. Data for visitor sessions will be captured passively through MHA’s website analytics system. Consistent with prior MHA research, a session is defined as activity on the MHA website from a unique IP address, bounded by 30 minutes of inactivity. Visitors with ≥30 minutes of inactivity are considered to have disengaged.

### Trial Design

#### Overview

This study uses an iterative series of MRTs to refine and test engagement strategies embedded within the MHA Online Screening Program. This approach is consistent with optimization frameworks that facilitate continual improvement of intervention components through successive trials [36]. In this study, eligible MHA visitors (henceforth, participants) may be randomized at 2 prespecified decision points embedded within the usual visitor flow of the MHA Online Screening Program, which could include progression through the PHQ-9 depression screening test, an optional postscreening survey that collects participant information (eg, demographics), screening results, and targeted Next Steps Content Pages. The first decision point will be the Screening Results Page, where MHA visitors are typically shown information about their PHQ-9 score and provided with a set of recommended resources as “Next Steps”. The second decision point will occur when participants navigate to a targeted Next Steps Content Page from the Screening Results Page. These 2 decision points represent critical moments where MHA visitors are at risk of disengagement.

**Figure 1.**
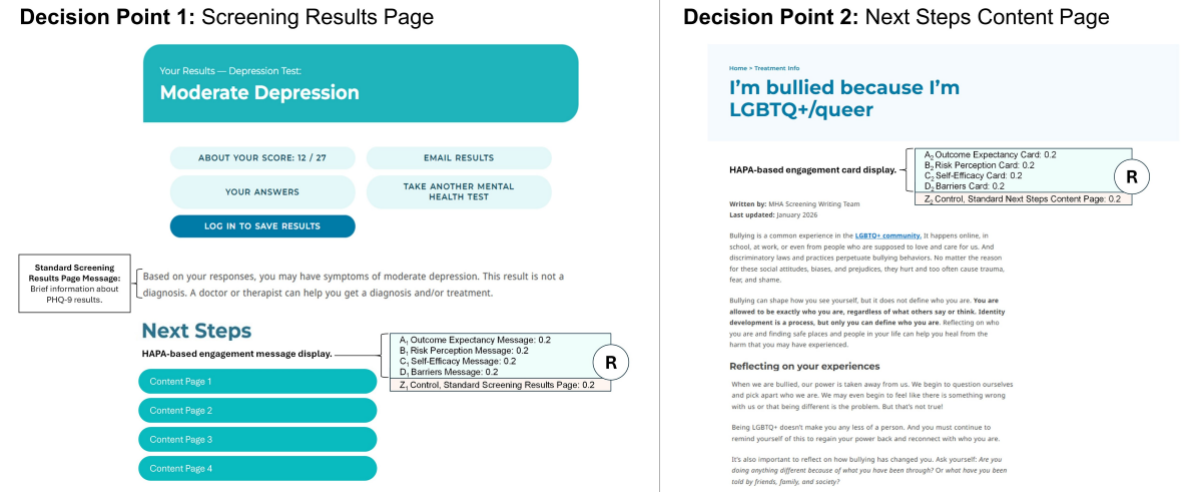
Engagement strategy delivery and randomization within the Mental Health America (MHA) website.

Distinct engagement strategies were designed for placement at the 2 decision points on the MHA website, including the Screening Results Page and the targeted Next Steps Content Pages. At each decision point, participants may be randomized to receive no engagement strategy (Z*_t_*) or an engagement strategy targeting 1 of 4 HAPA behavioral determinants, including outcome expectancy (A*_t_*), risk perception (B*_t_*), self-efficacy (C*_t_*), or SGM-specific barriers to engagement (D*_t_*) (Figure 1). Primarily, this series of MRTs will test the effect of delivering HAPA-based engagement strategies on proximal engagement compared to standard website content (ie, MHA pages without embedded engagement strategies).

#### Engagement Strategy Refinements Across MRT Iterations

Initial comparisons will be conducted across all available strategies. Over the study period, the set of strategies included in randomization may be refined based on accumulating evidence related to the performance of strategy content, delivery format, or timing. This iterative approach has previously been used to optimize engagement with DMH interventions, including on MHA [37], where strategies are refined and reevaluated in subsequent MRTs [40]. In this study, any engagement strategy refinements will be determined between MRT iterations and informed by findings from preceding MRTs. Refinements between MRT iterations will follow prespecified decision rules developed in consultation with MHA that balance optimization opportunities (eg, removing underperforming strategy variations within HAPA behavioral determinants or restricting to the highest-performing strategy variation within HAPA behavioral determinants) with feasibility and maintenance of a consistent MRT structure, including eligibility criteria, decision points, randomization to receive no engagement strategy (comparator) or an engagement strategy targeting HAPA behavioral determinants (intervention), and outcome measures. These refinements will be documented using the Framework for Reporting Adaptations and Modifications-Enhanced, which logs when the modification was made, what was modified, and the rationale for the modification, among other factors, to ensure transparency and analytic consistency [41]. No modifications to intervention content or randomization procedures are anticipated within an ongoing MRT unless they are necessary to address implementation errors identified through quality assurance (QA) monitoring. This approach supports optimization through efficient, progressive refinement of displayed strategies over the study period and may enable more direct comparisons among top-performing strategies while maintaining a consistent experimental framework across the series of MRTs. In alignment with similar studies, 3 to 4 MRTs are anticipated within the series, with the exact number of iterations depending on opportunities for continued strategy refinement and study time constraints [40]. Identifying the most effective strategies and delivery points will ultimately facilitate scalable implementation within the broader MHA website.

#### Eligibility Criteria

MHA visitors will be eligible for the trial if they complete the PHQ-9 depression screener in English and indicate on the optional postscreening survey that they (1) live in the United States, (2) are aged ≥14 years, and (3) identify as LGBTQ+ (lesbian, gay, bisexual, transgender, queer, and others).

#### Intervention and Comparator

Prior findings indicated that HAPA’s immediate behavioral determinants were associated with engagement behaviors (eg, time on resources, number of resources visited, and types of resources visited) among MHA’s SGM visitors. In this trial, engagement strategies targeting these behavioral determinants, delivered at key decision points, are hypothesized to increase proximal engagement by addressing participants’ outcome expectancies, perceived risks, self-efficacy, and SGM-specific barriers to engaging with DMH resources. Engagement strategies are designed to enhance participants’ beliefs that the Next Steps resources are relevant, helpful, and worth trying (target: outcome expectancy); strengthen feelings of safety and trust (target: perceived risk); increase confidence in their ability to use the resources (target: self-efficacy); and reinforce feelings of pride or support (target: SGM-specific barriers to engagement), thereby increasing proximal engagement relative to standard website content. To activate these mechanisms, engagement strategies incorporate evidence-based behavioral design elements, including authority cues, social proof, normative feedback, goal-gradient effects, and visually salient interface features (eg, highlighted calls to action) [42-44].

On the Screening Results Page, strategies tested in the initial MRT will be delivered as inline engagement messages designed to reinforce an immediate behavioral determinant to nudge participants to select their Next Step after viewing their PHQ-9 screening results. On the Next Steps Content Pages, strategies tested in the initial MRT will be delivered as engagement cards embedded within the existing page layout. Based on the preliminary work, an initial bank of HAPA-informed engagement strategy variations was developed. The bank includes 12 engagement message variations and 8 engagement card variations. Each variation was designed to target 1 of 4 HAPA behavioral determinants. During the intervention design process, HAPA behavioral determinants were operationalized based on how each manifested for SGM visitors, specifically (eg, risk perception: safety and trust concerns and SGM-specific barriers to engagement: stigma). For each determinant, 3 message variations were developed for the first decision point, and 2 card variations were developed for the second decision point. All variations within each determinant differ in wording but are unified by their focus on influencing the same behavioral determinant while maintaining consistent visual placement and interface design.

**Figure 2.**
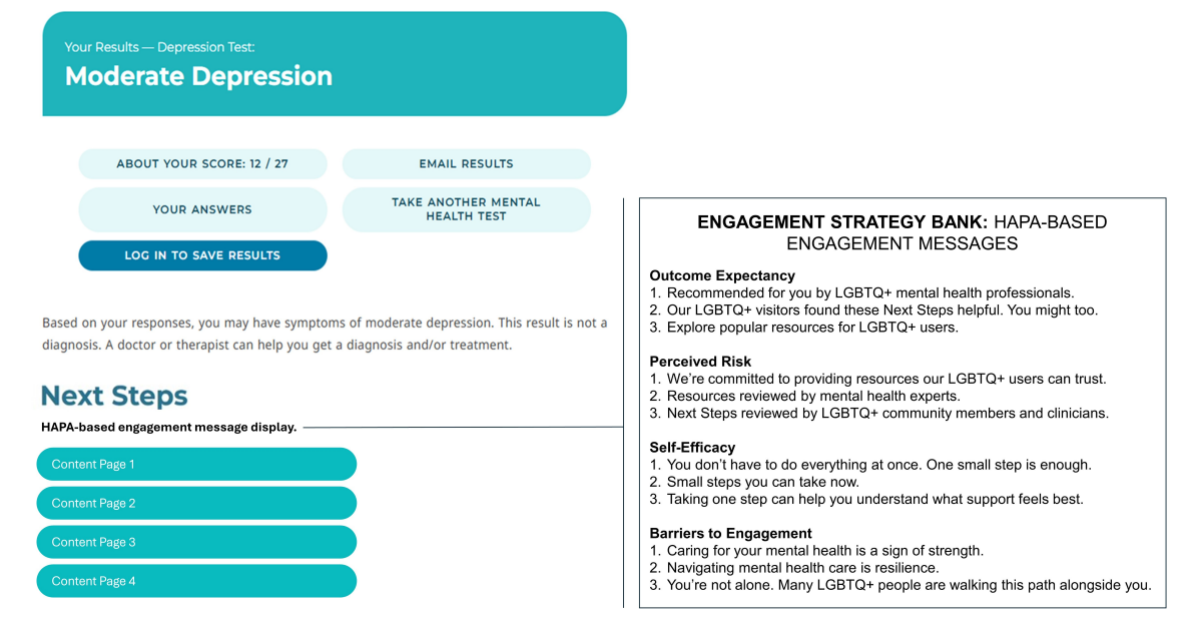
Health Action Process Approach (HAPA)–based engagement messages for the Screening Results Page.

On the Screening Results Page, participants will be randomized to see a brief inline engagement message targeting 1 of the 4 HAPA behavioral determinants or MHA’s standard Screening Results Page. At this initial decision point, engagement messages are designed to quickly reinforce engagement behaviors while minimizing cognitive burden at a high-friction transition point where most visitors disengage immediately after viewing their screening results. HAPA-based engagement messages that will be delivered at the first decision point in the initial MRT are displayed in Figure 2.

**Figure 3.**
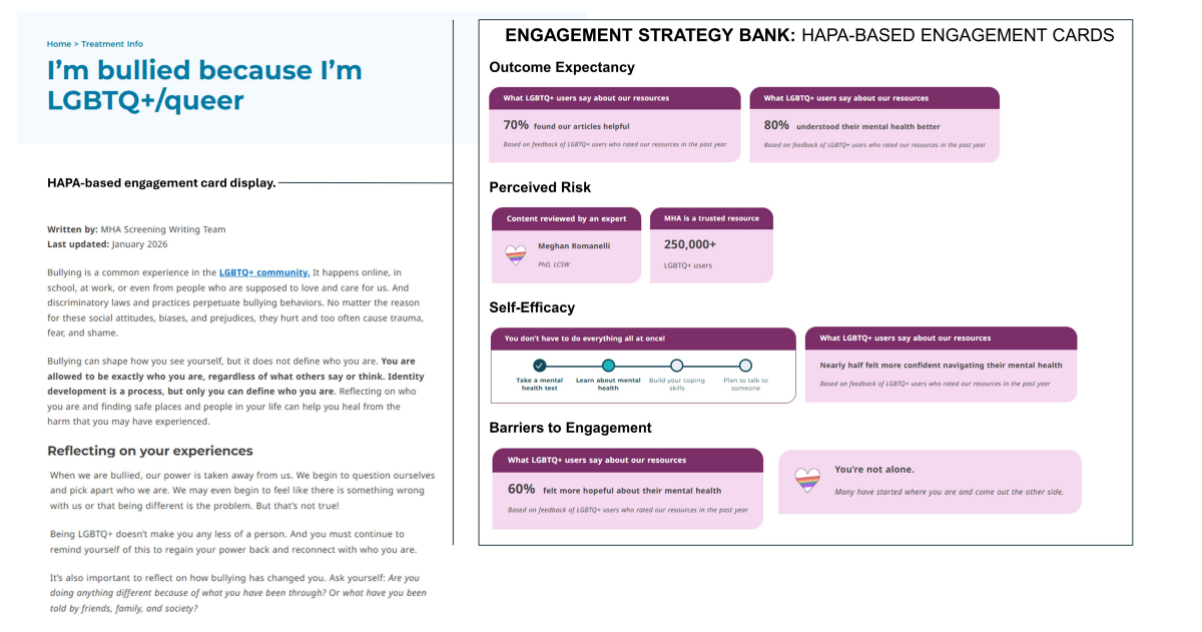
Health Action Process Approach (HAPA)–based engagement cards for the Next Steps Content Pages.

Once a participant initiates engagement by selecting one of the targeted Next Steps Content Pages, they may be randomized to see an engagement card embedded directly within the content page layout that targets 1 of the 4 HAPA behavioral determinants or MHA’s standard page design. These cards function as small user interface components that visually stand out but do not interrupt navigation. Cards are designed to provide a more visible but nondisruptive nudge to reinforce continued engagement. Each card will be embedded within the website interface and can be read without redirecting participants or requiring them to leave the page. HAPA-based engagement cards that will be delivered at the second decision point in the initial MRT are displayed in Figure 3.

#### Measures

##### Outcomes

Proximal engagement outcomes will be measured following each randomization. All engagement measures will be captured using server access logs, an objective measure of website browsing behavior, and bounded by 30 minutes of inactivity.

##### Primary Engagement Outcomes

At decision point 1 (Screening Results Page), the primary proximal outcome is initial engagement, which is defined as a binary indicator of whether a participant clicks to one of the targeted Next Steps Content Pages displayed on the Screening Results Page following randomization (yes or no). If a participant does not navigate to one of the targeted Next Steps Pages before leaving the MHA website or before the session ends due to 30 minutes of inactivity, then the outcome will be coded as “no.”

At decision point 2 (targeted Next Steps Content Pages), the primary proximal outcome is sustained engagement with MHA resources, which is defined as a binary indicator of whether a participant clicks to any additional substantive content page on the MHA website following randomization (yes or no). If a participant does not navigate to another content page before leaving the MHA website or before the session ends due to 30 minutes of inactivity, then the outcome will be coded as “no.”

##### Exploratory Engagement Outcomes

Exploratory proximal engagement outcomes are (1) total time (in seconds) spent on additional MHA content pages beyond the Screening Results Page and first targeted Next Steps Content Page; (2) total number of additional MHA content pages visited beyond the Screening Results Page and first targeted Next Steps Content Page; and (3) response, if available, to outcome questions displayed on Next Steps Content Page (eg, Was this helpful? Did this article help increase your knowledge and understanding of mental health? Did this article help you feel more confident in managing your mental health? and Did this article help you feel more hopeful about your mental health?).

For participants who click to another content page at decision point 2, specifically, sustained engagement with the targeted Next Steps Content Page will also be measured as total time (in seconds) spent on the page prior to navigating to the next page. For these participants, time on the targeted Next Steps Content Page can only be calculated when a subsequent navigation event occurs, as duration cannot be reliably computed when the Next Steps Content Page is the final page in a session.

##### Moderators and Covariates

All moderator and covariate measures will either be derived from server access logs or collected through MHA’s standard screening and optional postscreening survey.

##### Time-Varying Moderators

The number and type of MHA pages visited prior to a decision point will be treated as a time-varying moderator, as the value may differ depending on how much content a participant has accessed earlier in the session. These indicators capture the participant’s current engagement state at the moment the engagement strategy is delivered and allow examination of whether the effect of the strategy differs depending on earlier browsing behavior.

##### Time-Invariant Moderators

Sexual orientation, gender identity, age, baseline symptom severity (ie, PHQ-9 score), and participants’ stated postscreening intentions for using MHA (eg, reading articles, try a free self-help tool, and do nothing) will be treated as time-invariant moderators.

##### Covariates

Race or ethnicity and income will be treated as control variables.

### Sample Size

#### Overview

MRTs are often more statistically efficient than traditional randomized trial designs because they leverage both between-subject and within-subject contrasts of proximal outcomes. However, the efficiency gains of an MRT depend on the number of decision points contributed per participant. The current MRTs will include 2 decision points within a single visitor session on the MHA website. This design provides fewer within-person contrasts than high-frequency MRTs (eg, daily notification studies), as each participant can contribute at most 2 randomizations across the session. Consequently, statistical power in this study is primarily driven by the between-subject sample size rather than the repeated within-person contrasts. This structure reflects constraints of the intervention context. The current trial is a web-based, naturalistic engagement study that embeds randomizations within the existing ecosystem of a real-world DMH platform, where pull intervention delivery must align with the typical website navigation flow rather than repeated daily push notifications.

The minimum sample size was calculated for MRTs with binary proximal outcomes [36,45-47]. Calculations assumed 80% power, a 2-sided α of .05, and a treatment randomization probability of 0.80 for the primary pooled analysis (ie, probability of being assigned any HAPA-based engagement strategy vs control at a given decision point). An expected availability of 0.70 was used in the calculation. This reflects the expected availability averaged over time [46], including full availability at decision point 1 (as all eligible participants view the Screening Results Page) and a conservatively lower availability at decision point 2 (as preliminary analyses indicated that under standard conditions, most MHA visitors bounce after receiving their screening results, with fewer navigating to content pages). Participants who do not navigate to a Next Steps Content Page are not available for randomization at decision point 2. Under the assumption of a constant success probability under the null of 0.30 (a conservative estimate given that 63% of visitors leave the MHA website after viewing screening results) and a proximal treatment effect of 1.20 on the relative risk scale, an estimated 2300 participants will be required to detect a marginal causal excursion effect with 80% power. These assumptions reflect engagement patterns observed in prior analyses of MHA’s site use and account for the time-bounded, single-session nature of the intervention. Given the volume of visitors to MHA’s Online Screening Program, it is possible that the final sample size will exceed this minimum requirement. However, final enrollment numbers will ultimately reflect the naturalistic rate at which eligible visitors access MHA’s Online Screening Program throughout the duration of the study. Importantly, the study uses an iterative series of MRTs in which successive MRTs are conducted and engagement strategies are refined between iterations. Each MRT iteration requires approximately 2300 participants to detect an effect within the primary pooled analysis. To power secondary analyses comparing specific HAPA behavioral determinants against control or specific message or card against control, each MRT iteration will need a larger sample. These analyses will be interpreted as exploratory.

#### Recruitment

Given the real-world setting of this study and focus on naturalistic engagement as the primary outcome, no active recruitment or retention procedures will be implemented.

#### Intervention Assignment

##### Sequence Generation

As there will be no active recruitment for this trial, eligibility assessment will occur automatically following a visitor’s completion of the PHQ-9 depression screening and optional questions within MHA’s Online Screening Program (see *Eligibility Criteria* section).

Eligible participants will encounter 2 randomization decision points embedded within the usual flow of the Online Screening Program. The first decision point occurs on the Screening Results Page, where participants receive their PHQ-9 score, brief information about their score, and a set of 3 to 4 targeted Next Steps Content Pages. At this point, participants will be randomized with equal probability to either receive no engagement strategy (ie, standard Screening Results Page) or an engagement message targeting 1 of 4 HAPA behavioral determinants (ie, outcome expectancy, perceived risk, self-efficacy, and SGM-specific barriers to engagement). Here, engagement strategies are drawn from a bank of messages developed to operationalize each determinant.

The second decision point will occur when participants navigate to a targeted Next Steps Content Page from the Screening Results Page. At this point, participants will again be randomized with equal probability to either receive no engagement strategy (ie, standard Next Steps Content Pages) or an engagement card targeting 1 of the same 4 HAPA behavioral determinants. Here, engagement strategies are drawn from a bank of cards developed to operationalize each determinant.

Randomizations will be independent across decision points. Randomization probabilities will be fixed across participants and do not depend on prior randomizations, engagement outcomes, or participant characteristics.

##### Allocation Concealment Mechanism and Blinding

Participants will not be explicitly informed that engagement strategies are being experimentally manipulated due to the naturalistic design of the trial and the waiver of consent. Participants will experience engagement strategies as part of the MHA interface and will not be notified of their assigned condition, the existence of multiple conditions, or randomization schedules. Engagement strategies will not alter navigation options or restrict access to content.

##### Implementation

Randomization with equal allocation across conditions will be implemented at each decision point through MHA’s web infrastructure. The randomization logic was developed in collaboration with MHA’s research team and will undergo QA testing prior to the launch of each MRT iteration to ensure correct allocation probabilities and independence across decision points. Delivery of engagement strategies will occur passively within the existing website interface and does not require additional participant actions beyond normal site navigation.

### Data Analysis Plan

#### Primary Aim Analysis

Analyses will estimate the marginal causal excursion effects of delivering engagement strategies on proximal engagement with MHA content. Data will be analyzed using the weighted centered least squares estimator, which provides consistent estimates of causal excursion effects when including time-varying factors that may be influenced by prior intervention exposure [36,45,48]. Analyses will examine whether, on average, across decision points, engagement strategies (A*_t_*-D*_t_*) increase proximal engagement with targeted MHA content relative to the control (Z*_t_*). A log-linear model will be used, as the primary proximal outcome is binary (engaged vs not engaged with targeted content). For the primary analysis, all HAPA-based engagement strategies (A*_t_*-D*_t_*) will be pooled so that the estimated marginal causal excursion effect represents the average effect of delivering any engagement strategy compared with the control (Z*_t_*). Effects will be expressed as the log of a risk ratio (ie, the probability of engagement when an engagement strategy is delivered relative to the probability of engagement when the control is delivered at a decision point). To examine whether intervention effects differ across the 2 decision points, models will include an interaction between intervention assignment and a decision point indicator. Baseline characteristics (eg, sexual orientation, gender identity, age, baseline PHQ-9 score, and intentions for using MHA) and time-varying contextual factors (eg, number and type of pages visited prior to a decision point) will also be examined as potential moderators of proximal intervention effects by including interaction terms in the log-linear model. Models will control for race or ethnicity and income.

#### Secondary Aim Analysis

Secondary analyses will examine whether engagement strategies targeting distinct HAPA behavioral determinants differ in their relative effectiveness at increasing proximal engagement with targeted MHA content. Using the same weighted centered least squares estimation approach, models will compare the proximal effects of strategies targeting outcome expectancy (A*_t_*), risk perception (B*_t_*), self-efficacy (C*_t_*), and SGM-specific barriers to engagement (D*_t_*). These analyses will estimate marginal causal excursion effects for each strategy type. Additional analyses will examine whether variations of engagement strategies within the bank that target the same HAPA behavioral determinant differentially impact proximal engagement. These exploratory comparisons will be conducted by extending the primary regression models to include indicators for specific engagement strategy variations and estimating their relative causal excursion effects on proximal engagement outcomes. Exploratory analyses will also examine additional engagement outcomes (as described in the *Measures* section), including time spent on pages and the number of additional pages visited following exposure to intervention content.

#### Missing Data

Proximal outcomes at both decision points are defined using website event data and are expected to be largely complete. Missing proximal outcomes may occur due to both technical factors (eg, logging errors) and behavioral processes (eg, participants who disengage before a decision point are not available for randomization). Indicators required for eligibility assessment are expected to be complete, but some moderators and covariates may be missing as postscreening survey items are optional.

45 48

The primary analysis will use all available decision points with observed proximal outcomes (ie, complete-case analysis at each decision point), an approach consistent with recommendations for addressing missing data in MRTs [49]. If missing data are ≤10%, results will be interpreted with minimal concern for bias. If missingness exceeds this threshold, patterns of missing data will be summarized at each decision point and compared across experimental conditions and observed participant characteristics. Exploratory analyses will examine whether intervention effects differ depending on patterns of missingness.

### Ethical Considerations

#### Institutional Review Board Approval

This trial protocol was reviewed by the single institutional review board (sIRB) at the University of Washington and approved on December 3, 2025 (STUDY00017726). The QT-Digital Mental Health Engagement study is considered a multisite clinical trial, as MHA is involved in the following research activities: (1) performing research procedures; (2) administering study interventions; and (3) obtaining, using, or analyzing identifiable data. The external Data Safety and Monitoring Board was provided with protocol documents prior to the initiation of data collection and did not identify any foreseeable risks. Any major protocol modifications will be reviewed by the sIRB and the Data Safety and Monitoring Board and updated on the ClinicalTrials.gov registration.

#### Waiver of Consent

A waiver of consent was approved by the sIRB to facilitate the examination of naturalistic engagement. This waiver was supported by (1) MHA’s privacy policy, which informs visitors who use the site’s screening tools that deidentified data may be shared with universities for research and quality improvement purposes (Multimedia Appendix 1); and (2) the study’s minimal risk classification, as participants can continue to access MHA’s content across all conditions. In this study, formal consent procedures may also prevent naturalistic engagement. Prior research has found that participants recruited explicitly for DMH research engage differently than users who access DMH and are not identified as research participants [50]. Although participants will be randomized to different engagement strategies, these strategies introduce very minimal risk and are intended to optimize engagement with MHA’s available DMH resources.

#### Adolescent Participants

Along with the overall waiver of consent, a waiver of parental consent was approved for participants aged 14 to 17 years, as the protocol was designed for a subject population (here, SGM adolescents seeking readily available DMH resources via MHA’s platform) for which parental permission is not a reasonable requirement to protect this study subsample (45 CFR 46.408(c)). Generally, adolescents represent approximately 59% of MHA’s SGM visitors. In alignment, as of April 13, 2026, 52% (2004/3878) of study participants were SGM visitors aged 14 to 17 years. Including this user population is critical to understanding and optimizing the engagement behaviors of MHA’s SGM visitors, generally, and for this priority subpopulation. Adolescent-parental relational and communication issues are often cited as the main barriers to mental health care for SGM adolescents [51]. Besides disrupting natural engagement, requiring parental consent may cause safety concerns for those not ready to disclose their sexual or gender identity [52-54] or for those seeking DMH services due to relational concerns with their parents. The lower cutoff age of 14 years was chosen, as these adolescents represent high school–aged youth whom past research shows have similar health literacy as adults [55] and sufficient maturity and rational decision-making to self-consent to health and mental health care [56].

#### Risk Mitigation

MRT participants will be subject to minimal levels of risk, no greater than if they were to use the MHA platform without enrollment in this study. Participants reached and will engage with the MHA platform and Online Screening Program of their own volition. Although the study outcome is engagement, it is expected that most participants will have mental health symptoms or diagnoses. All MHA visitors have access to contact information for “warmlines” for each of the 50 states and phone and text crisis lines (eg, suicide and interpersonal violence), including those tailored to SGM populations. MHA visitors displaying any heightened level of risk for suicide (eg, anyone who responds “several days,” “more than half the days,” or “nearly every day” on the PHQ-9) are immediately prompted with the message “If you need immediate help, you can reach the Suicide & Crisis Lifeline by calling or texting 988 or using the chat box at 988lifeline.org. You can also text “MHA to 741-741 to reach the CrisisText Line. Warmlines (embedded link to warmline resources) are an excellent place for non-crisis support.” These mechanisms remain in place for study participants.

#### Risk Mitigation

Along with the overall waiver of consent, a waiver of parental consent was approved for participants aged 14 to 17 years, as the protocol was designed for a subject population (here, SGM adolescents seeking readily available DMH resources via MHA’s platform) for which parental permission is not a reasonable requirement to protect this study subsample (45 CFR 46.408(c)). Generally, adolescents represent approximately 59% of MHA’s SGM visitors. In alignment, as of April 13, 2026, 52% (2004/3878) of study participants were SGM visitors aged 14 to 17 years. Including this user population is critical to understanding and optimizing the engagement behaviors of MHA’s SGM visitors, generally, and for this priority subpopulation. Adolescent-parental relational and communication issues are often cited as the main barriers to mental health care for SGM adolescents [51]. Besides disrupting natural engagement, requiring parental consent may cause safety concerns for those not ready to disclose their sexual or gender identity [52-54] or for those seeking DMH services due to relational concerns with their parents. The lower cutoff age of 14 years was chosen, as these adolescents represent high school–aged youth whom past research shows have similar health literacy as adults [55] and sufficient maturity and rational decision-making to self-consent to health and mental health care [56].

#### Confidentiality

MHA collects engagement data and optional demographic survey responses as part of standard operations. MHA’s privacy policy provides visitors with information about how data are collected, stored, and shared (Multimedia Appendix 1). IP addresses are recorded and stored indefinitely in MHA’s server access logs but are not processed to identify visitors. MHA will have access to participant identifiers, including IP addresses and ZIP codes or email addresses, if provided by visitors as part of the optional demographic survey. UW researchers will not have access to any of these direct identifiers as they are removed from datasets before transmission from MHA to UW via a secure Box account. IP addresses, specifically, are hashed using an MD5 algorithm. This process creates an indirect identifier that cannot be reversed to recover the original IP address. MHA’s procedures for data cleaning, including deidentification procedures, have been published elsewhere [37]. The deidentified data will be stored on UW servers in accordance with the University’s data security requirements.

## Results

This study received funding from the National Institute of Mental Health (NIMH) in June 2023 (K01MH131795). Enrollment for the initial MRT began on March 12, 2026. As of April 13, 2026, a total of 3878 participants have been enrolled. Data collection for all MRT iterations is projected to end in July 2026. Prior to each MRT iteration, QA testing will be conducted live on the MHA website to monitor for programming and protocol execution errors. Data generated during QA periods will be retained and included in the analytic dataset if no substantive issues are identified. If any issues affecting randomization, intervention delivery, or outcome capture are detected, QA testing will continue until the issues are resolved. Data collection for analysis will then begin following a subsequent period of confirmed error-free operation. QA monitoring for programming and protocol execution errors will continue throughout the data collection period.

## Discussion

This study will evaluate the delivery of brief, HAPA-informed engagement strategies within MHA’s Online Screening Program and estimate their proximal effects on naturalistic engagement among SGM visitors. By embedding an iterative series of MRTs into a high-volume, real-world platform, the study is designed to test whether brief strategies integrated into the standard website experience increase initial and sustained engagement. The results will also support analyses that can inform iterative refinement of engagement strategies and the development of decision rules for when and how to deliver engagement supports in real-world DMH settings.

Several limitations should be considered when interpreting study findings. First, generalizability may be limited because trial eligibility depends on completion of an optional postscreening survey. The analytic sample may differ from otherwise eligible SGM visitors who do not complete demographic questions. Second, engagement outcomes are measured using website metadata within sessions bounded by inactivity, and the study cannot reliably link individuals across multiple visits because MHA does not require visitors to create accounts to access their free, anonymous resources. Consequently, we will be unable to examine longer-term engagement trajectories, repeated visits, or actions taken outside the website. Relatedly, proximal engagement outcomes such as clicks and time on page capture observable behavioral interactions but do not fully reflect experiential aspects of engagement, including attention, perceived relevance, or affective response. These measures also do not capture whether engagement ultimately leads to improvements in mental health outcomes. Third, sessions are defined using IP-based identifiers, which may not always distinguish unique individuals when multiple visitors access the site from shared networks or households. However, this approach aligns with prior MHA research and provides a practical method for measuring engagement at scale. Fourth, because engagement strategies are embedded within existing webpages, exposure cannot be guaranteed for every eligible participant. For example, if a participant does not scroll to the portion of the page where a HAPA-based message or card is embedded, estimated effects may be attenuated. Finally, because engagement strategies must be integrated into MHA’s existing visitor flow, the number and placement of decision points, frequency of strategy delivery, and types of intervention components delivered are constrained by feasibility within MHA’s existing site architecture and technical infrastructure. Although participant flow is largely predictable through the Screening Results and initial Next Steps Content Pages, navigation becomes too diffuse thereafter to feasibly implement and evaluate intervention components across all subsequent pages. The current MRT design only includes 2 decision points within a single browsing session. As a result, the degree of repeated randomization is limited compared with higher-frequency MRTs and may reduce the ability to estimate time-varying intervention effects. This structure reflects pragmatic constraints of embedding experimentation within a large, publicly available DMH website, where repeated prompts could be burdensome or disruptive to naturalistic use. Under similar constraints, the engagement strategies are limited by their design as pull components, and their effectiveness may depend on the participants’ mindset and behavior (eg, belief that MHA’s Next Step resources are needed) [36]. However, these trial and engagement strategy design choices all reflect feasibility constraints identified through the Discover, discover, Design, Build, and Testdesign, build, and test process and are consistent with the study’s aim to evaluate implementation within a real-world setting.

Despite these limitations, this protocol advances DMH engagement research by testing theory-based, SGM-relevant engagement strategies in a real-world setting where disengagement can occur within seconds. Results will identify which strategies show promise for improving naturalistic engagement at key drop-off points, whether effects differ across subgroups (eg, demographics, depression subtypes, and behavioral intentions) and contexts, and which design choices are feasible within MHA’s infrastructure. This information will guide refinement of message and card content and decisions about where additional decision points may be needed. Final study results and implications will be summarized and presented to MHA and reported to the study registry and sponsor. Results will also be disseminated to the wider scientific community through peer-reviewed publications and conferences. Although tested within MHA’s Online Screening Program, the approach of embedding brief engagement strategies at empirically identified drop-off points may be applicable to other self-guided DMH platforms that face similar challenges with user engagement. Longer-term work should examine whether proximal engagement gains translate to meaningful downstream behaviors, including use of additional resources, help-seeking, and symptom change.

## Supplementary material

10.2196/97126 Multimedia Appendix 1 Mental Health America privacy policy.

10.2196/97126 Checklist 1 SPIRIT checklist.

## Abbreviations

DMH: digital mental health
HAPA: Health Action Process Approach
LGBTQ+: lesbian, gay, bisexual, transgender, queer, and others
MHA: Mental Health America
MRT: microrandomized trial
NIMH: National Institute of Mental Health
PHQ-9: Patient Health Questionnaire-9
QA: quality assurance
SGM: sexual and gender minority
sIRB: single institutional review board
SPIRIT: Standard Protocol Items: Recommendations for Intervention Trials
