# Supplementary material for: Optimizing Engagement with Digital Mental Health Resources Among Sexual and Gender Minority Users: Protocol for a Series of Microrandomized Trials
Source: JMIR Res Protoc. 2026 Jun 30;15:e97126. doi: 10.2196/97126 (PMC13318396; doi:10.2196/97126)
Supplement: Multimedia Appendix 1 [file resprot-v15-e97126-s001.pdf]

Privacy Policy

mhanational.org/privacy-policy

Mental Health America Website: Privacy Policy

Effective Date: May 11, 2021

This Privacy Policy describes the types of personal information collected by Mental Health America, Inc. ("MHA" or "Mental Health America") how we use such information, and to whom and under what circumstances we may disclose it. This Privacy Policy applies to MHA's website, www.mhanational.org (the "Site") and any personal information obtained when you call, email, or otherwise communicate with MHA. By accessing the Site or otherwise interacting with us, you agree to this Privacy Policy. We can modify this Privacy Policy at any time, and will post the current version on the Site. We encourage you to periodically review our Privacy Policy to stay informed about how we are using the information we collect.

Information We Collect When You Visit the Site

For each visitor to our Site, we may collect information such as the domain name of the website the visitor came from, the e-mail addresses of those who post messages to our bulletin board (if given), the e-mail addresses of those who communicate with us via e-mail, aggregate information on what pages visitors access or visit, information volunteered by a visitor, such as survey information, participation in chats or discussion boards, and/or site registrations. MHA does not sell or rent your information for commercial purposes.

With respect to Tell-A-Friend: If a user elects to use our referral service to inform a friend about our Site, we ask them for the friend's name and email address. Mental Health America's Advocacy Network will automatically send the friend a one-time email inviting them to visit the Site. This information is stored for the sole purpose of sending this one-time email.

With respect to SmallStepsSMS: If a user elects to use our text messaging service to receive mental health education and skills training, we will ask them for their phone number and some demographic information (for example age). Phone numbers and any other identifying information are stored by Audacious Software, who is partnering with MHA, for the sole purpose of sending text messages. Phone numbers are encrypted so that they cannot be traced back to the user. Demographic information will be used for the purpose of personalizing the text messages a user receives.

Those who score above threshold on the PQ-B will have the opportunity to participate in a research project in collaboration with researchers at Columbia University and sponsored by the National Institute of Mental Health. The goals of this project develop and test online and text-based strategies to support help-seeking. Youth who score positive for psychosis risk will be

randomized to test the effectiveness of different help-seeking advancement strategies. Participants will also be able to interact with research staff over text and video chat, which is being supported by Twilio, Spoke Phone and Dexterous. Participants may choose to schedule a convenient time to chat or meet with a member of the research team virtually using Acuity. Data collected from texting interactions will include anonymous meta data (number of texts initiated, number of texts responded to, timing of texts). Data collected from text and video interactions will also include aggregated language data for natural language processing.

We may collect this information and use it for marketing and communications purposes, internal review, research, and to improve the content of our Web page, but personally identifiable information is generally not shared with other organizations for commercial purposes.

Cookies: To help make our Site more responsive to the needs of our visitors, we invoke a standard feature found in browser software, called a “cookie”, to assign each visitor a unique, random number, a sort of anonymous user ID that resides on your computer. The cookie identifies the computer that a visitor uses to access the Site, but cannot access or read information from your computer’s hard drive. You can delete or disable cookies at any time by changing the preferences in most web browsers.

For our internal purposes, we gather date, time, browser type, navigation history and IP address of all visitors to our site. We use this information for our internal security audit log, trend analysis and system administration, and to gather broad demographic information about our user base for aggregate use. We collect more information with our Screening Tools, as discussed below.

Screening Tools

If you use our screening tools on the Site, you provide us with personal information regarding your mental health symptoms, such as depression or anxiety. We also collect voluntary demographic information, including age range, gender, race/ethnicity, household income, state, and zip code. We endeavor to store screening data in a way that cannot be used to identify you. For example, IP addresses associated with a particular screening session are protected by one-way hash so that they cannot be traced back to the original IP address. We may provide deidentified or aggregated data to universities, health providers or others for research purposes, in all cases taking reasonable steps to ensure that such data cannot be associated with any particular individual.

Those who score above threshold on the PQ-B will have the opportunity to participate in a research project in collaboration with researchers at Columbia University and sponsored by the National Institute of Mental Health. The goals of this project develop and test online and text-based strategies to support help-seeking. Youth who score positive for psychosis risk will be randomized to test the effectiveness of different help-seeking advancement strategies. Participants will also be able to interact with research staff over text and video chat, which is being

supported by Twilio and Dexterous. Data collected from texting interactions will include 1) anonymous meta data (number of texts initiated, number of texts responded to, timing of texts) and 2) aggregated language data for natural language processing.

Donor Information

Mental Health America treats donor information that is collected offline in the same manner as it treats information collected online. Donor payment information may be collected through third-party processing services. Mental Health America does NOT endorse companies, products or services and strictly prohibits the suggestion of endorsement, recommendation, or superiority of one company, product or service over another company, product or service.

Other Ways We May Disclose Your Information

We may also disclose information collected from and about you as follows: (1) to our related companies and service providers, to perform a business, professional or technical support function for us; (2) to our marketing partners, advertisers or other third parties, who may contact you regarding the mission and opportunities with MHA; (3) as necessary if we believe that there has been a violation of the Site Terms of Use or of our rights or the rights of any third party; (4) to respond to legal process (such as a search warrant, subpoena or court order) and provide information to law enforcement agencies or in connection with an investigation on matters related to public safety, as permitted by law, or otherwise as required by law; and (5) in the event that our MHA or substantially all of its assets are acquired, your personal information may be one of the transferred assets. We may also disclose your personal information with your express consent.

Please note that if you voluntarily submit any personal information for posting on the Sites, such as a review or a blog post, the information becomes publicly available and can be collected and used by others, so you should use care before posting information about yourself online.

Links to Other Web Sites

When Mental Health America's Site includes links to other sites, MHA does not assume any responsibility or liability for any communications or materials available at the sites to which it links, including responsibility or liability for their accuracy. No link on Mental Health America's Site is a referral or endorsement of either the linked-to entity or any product or service.

Privacy Policy Changes

If our information practices change at some time in the future, we will post the policy changes to our Site to notify you of these changes and may provide you with the ability to opt out of these new uses. If you are concerned about how your information is used, you should check back at our Web site periodically.

Security Information

With respect to security: We endeavor to make sure that our Site and services provided by MHA protect personal information that we receive are secure. When we transfer and receive certain types of sensitive information such as financial information, we redirect visitors to a secure server and will notify visitors through a pop-up screen on our Site. However, no Site or online transaction is completely and perfectly secure, and you should exercise care and judgment in ensuring that you follow good security practices, including accessing our Site through a secure device and internet connection.

All sensitive information provided by individuals to Mental Health America is considered confidential, unless otherwise noted. Information is never sold or traded. If Mental Health America wishes to use any confidential information provided, the provider will be contacted, and a release form outlining its use will be provided. Individuals retain the right to request Mental Health America remove their information from all mailings, both online and on paper at any time, and Mental Health America will endeavor to remove the information in a reasonable manner, but delays between any such request and the discontinuation of related mailings can occur.

Your Options Concerning Personal Information

We provide all visitors to our Site with the ability to make certain requests regarding their personal information. For example, you can request:

- information regarding the categories and specific information we have collected about you;
- correction of information we have about you that is incorrect; and
- deletion of information we have collected about you.

Your exercise of these rights will have no adverse effect on the price and quality of our goods or services. When you exercise these rights and submit a request to us, we may verify your identity by asking you for your email address, telephone number, or other information. We also may use a third party verification provider to verify your identity.

Opt-Out for Email and Regular Mail: If you provide us with your email or postal address, we may contact you unless you explicitly opt out by calling us, mailing us, or emailing us at info@mhanational.org or by calling 800-969-6MHA.

To make any of these requests, please contact us as described below, under Contact Us.

Visitors from Outside the United States—Cross-Border Transfer

If you are visiting the Site from outside the United States, your information may be transferred to, stored and processed in the United States or other countries in accordance with this Privacy Policy. The data protection and other applicable laws of the United States or other countries may not be as comprehensive as those laws or regulations in your country or may otherwise differ from the data protection or consumer protection laws in your country. Your information may be available

to government authorities under lawful orders and law applicable in such jurisdictions. By using the Site and/or providing personal information to us, you consent to transfer of your information to our facilities as described in this Privacy Policy.

Contact Us

If you have questions or concerns about this Privacy Policy or how we collect and use personal information, you can contact us:

Email: info@mhanational.org

Telephone: 800-969-6MHA
